# The H-ATOMIC Criteria for the Etiologic Classification of Patients with Intracerebral Hemorrhage

**DOI:** 10.1371/journal.pone.0156992

**Published:** 2016-06-08

**Authors:** Joan Martí-Fàbregas, Luis Prats-Sánchez, Alejandro Martínez-Domeño, Pol Camps-Renom, Rebeca Marín, Elena Jiménez-Xarrié, Blanca Fuentes, Laura Dorado, Francisco Purroy, Susana Arias-Rivas, Raquel Delgado-Mederos

**Affiliations:** 1 Stroke Unit, Department of Neurology, Hospital de la Santa Creu i Sant Pau, IIB-Sant Pau, Barcelona, Spain; 2 Department of Neurology, Hospital Universitario La Paz, Madrid, Spain; 3 Hospital Universitari Germans Trias i Pujol, Badalona, Spain; 4 Hospital Universitari Arnau de Vilanova, Lleida, Spain; 5 Complejo Hospitalario Universitario de Santiago, Santiago de Compostela, Spain; Heinrich-Heine University, GERMANY

## Abstract

**Background and Purpose:**

There are no generally accepted criteria for the etiologic classification of intracerebral hemorrhage (ICH). For this reason, we have developed a set of etiologic criteria and have applied them to a large number of patients to determine their utility.

**Methods:**

The **H-ATOMIC** classification includes 7 etiologic categories: **H**ypertension, cerebral **A**myloid angiopathy, **T**umour, **O**ral anticoagulants, vascular **M**alformation, **I**nfrequent causes and **C**ryptogenic. For each category, the etiology is scored with three degrees of certainty: Possible_(3)_, Probable_(2)_ and Definite_(1)_. Our aim was to perform a basic study consisting of neuroimaging, blood tests, and CT-angio when a numerical score (SICH) suggested an underlying structural abnormality. Combinations of >1 etiologic category for an individual patient were acceptable. The criteria were evaluated in a multicenter and prospective study of consecutive patients with spontaneous ICH.

**Results:**

Our study included 439 patients (age 70.8 ± 14.5 years; 61.3% were men). A definite etiology was achieved in 176 (40.1% of the patients: Hypertension 28.2%, cerebral Amyloid angiopathy 0.2%, Tumour 0.2%, Oral anticoagulants 2.2%, vascular Malformation 4.5%, Infrequent causes 4.5%). A total of 7 patients (1.6%) were cryptogenic. In the remaining 58.3% of the patients, ICH was attributable to a single (n = 56, 12.7%) or the combination of ≥2 (n = 200, 45.5%) possible/probable etiologies. The most frequent combinations of etiologies involved possible hypertension with possible CAA (H_3_A_3_, n = 38) or with probable CAA (H_3_A_2_, n = 29), and probable hypertension with probable OA (H_2_O_2_, n = 27). The most frequent category with any degree of certainty was hypertension (H_1+2+3_ = 80.6%) followed by cerebral amyloid angiopathy (A_1+2+3_ = 30.9%).

**Conclusions:**

According to our etiologic criteria, only about 40% patients received a definite diagnosis, while in the remaining patients ICH was attributable to a single possible/probable etiology or to more than one possible/probable etiology. The use of these criteria would likely help in the management of patients with ICH.

## Introduction

The epidemiology, pathogenesis, prognosis, and response to treatment of patients with Intracerebral Hemorrhage (ICH) may differ depending upon the etiology. By analogy with ischemic stroke, an etiologic classification of ICH may improve the care for patients with ICH. About 80% of cases of ICH are primary ICH (65% attributed to arterial hypertension, 15% to cerebral amyloid angiopathy–CAA-) and the remaining 20% are considered secondary ICH [1]. However, there are no accepted criteria to attribute the etiology to a specific condition in an individual patient. Moreover, a patient may have more than one etiology, so a possible diagnosis or a probable one is more realistic than a definite one. The use of complementary examinations to improve the diagnostic accuracy is an undefined strategy [2]. A recent study [3] proposed an etiologic classification of ICH called SMASH-U. In this study no degrees of certainty were provided and a classification in more than one category was not allowed.

We believe that for progress in the understanding of ICH, there should be a set of criteria for the etiologic classification of ICH that considers the degrees of diagnostic certainty. To that end a minimum of complementary examinations is required and an assumption that some patients will share components of more than one etiologic category. We have developed a set of etiologic criteria (the H-ATOMIC criteria) and have applied them to a large number of patients in a multicenter and prospective study.

## Materials and Methods

The local Ethics Committee (Hospital de la Santa Creu i Sant Pau, Barcelona, Spain) approved the study. A signed consent from each patient or the legal representative was not deemed necessary by the Committee due to the absence of a change in the routine management of the patients and also that the data obtained were anonymous

### Description of the H-ATOMIC criteria

#### 1) Etiologic categories

According to the most frequent etiologic categories, the H-ATOMIC classification includes the following 7 subtypes of ICH: H = arterial Hypertension, A = CAA, T = Tumour, O = Oral anticoagulants (OA), M = arterio-venous Malformations and cavernoma, I = Infrequent causes associated to ICH, and C = unknown cause or Cryptogenic (by analogy with ischemic stroke classifications, this category is considered an etiologic subtype). The ICH to be classified is the one that constitutes current hospital admission. The only exception is CAA, where a previous lobar hematoma assists in the classification of the current event.

#### 2) Strategy of the etiologic work up

A prerequisite for the etiological classification is to perform a basic study as soon as possible after admission (preferably within the first 24 hours). An extended study is at the discretion of the clinician based on clinical evaluation and findings of the complementary explorations and neurologic status.

The basic study must include: 1) Neuroimaging: Cranial CT is the examination of choice for the diagnosis of ICH due to it is readily available, the results are highly specific, easy to interpret and rapidly obtained. Also, it is tolerated well by most patients. MRI is an acceptable alternative since its diagnostic accuracy is at least as high as CT [4, 5] although it is less available and is contraindicated in some patients; 2) Blood tests: They include cell counts (red and white cells and platelets); blood chemistry (sodium, potassium, blood glucose, urea, creatinine and transaminase levels) and blood coagulation (activated thromboplastin time, international normalized ratio and thrombin time); 3) Electrocardiogram and chest radiography. Although its diagnostic utility is low it is done in every acute stroke patient; 4) CT-angio or other angiographic modalities (MR-angio, conventional catheter angiography) when the Secondary Intracerebral Hemorrhage score [6] is >2. The SICH score includes the information from a non-enhanced CT, age, sex and relevant medical history (arterial hypertension, impaired coagulation) and ranges from 0 to 6; a SICH score >2 successfully predicts a given ICH patient’s risk of having an underlying vascular etiology. It is best to obtain the angiographic information as soon as possible (ideally immediately after the diagnostic CT) but the clinician must decide when the best moment is to perform this examination. However, the basic study is not considered complete in patients with a SICH score >2 until the angiographic study is completed. The absolute or relative contraindications of this examination should be taken into account also.

The etiologic classification may be made at discharge, or before if the patient dies during hospital admission. However, the final diagnosis may be delayed up to 3 months after ICH, if a follow-up neuroimaging study or any new relevant information changes the etiologic category within this period. Typical examples are a vascular abnormality (arterio-venous malformation, dural fistula) which needs a delayed angiography, a cavernomatous malformation which needs a follow-up MR or an ICH that is suspicious of harbouring a tumour and that will be better depicted by a follow-up CT or MR.

The extended etiologic work up may include any or all of the following: 1) Vascular imaging, when it is necessary to rule out an underlying vascular lesion that is suspected regardless of the SICH score. In some patients, more than one angiographic examination may be needed, and arterial as well as venous imaging (when venous thrombosis is suspected) may be advisable; 2) A MRI with gradient-echo sequence to depict microbleeds and siderosis that may be supportive of CAA, hypertensive angiopathy or both [7]; 3) A delayed neuroimaging or a repeated angiographic examination (that must be performed within the 3 months after the onset of ICH) when it is likely that an underlying lesion will be better detected after reabsortion of blood; 4) Transthoracic echocardiography and/or a blood pressure map for the diagnosis of prior arterial hypertension; 5) Quantitative or qualitative measurement of the use of illegal drugs in urine; 6) Cerebrospinal fluid analysis when vasculitis is suspected; 7) Biopsy or pathological examination of specimen obtained by surgical evacuations or at autopsy; 8) Screening for a primary cancer when metastasis is suspected; 9) Contrast-CT or contrast-MRI or PET when a primary tumour or metastasis is suspected.

#### 3) Degree of certainty of the etiologic classification

Each of the above etiologic categories is scored with a degree of certainty of Definite (1), Probable (2) and Possible (3). When more than one category is scored, the letters follow the order of the acronym H-ATOMIC. For example, T_2_O_2_ is correct but O_2_T_2_ is incorrect.

A patient can be scored as “definite” in only one etiologic category, because the category of '1' implies that there are no other '2' or '3' etiologies. For example, M_1_ means that a patient has a definite arterio-venous malformation but there is no possible or probable etiology regarding hypertension, CAA, tumour, OA or infrequent causes. A patient can be classified as '3' or '2' in one or more etiologic categories, provided no definite diagnosis has been made. For example, a patient scored as H_3_A_3_O_3_ means that the patient has possible hypertensive, CAA and OA-related ICH.

#### 4) Definitions of each etiologic category

H (Hypertensive ICH, see Table 1). Definite hypertensive ICH (H1). The following criteria must be fulfilled. 1) The patient had prior arterial hypertension. If the patient or family members do not know whether the patient was hypertensive prior to the ICH, this condition is fulfilled if electrocardiogram or transthoracic echocardiography show typical findings associated with left ventricle hypertrophy, or if arterial hypertension is demonstrated beyond 7 days after ICH (with at least two measurements above 140/90 mmHg in a patient not receiving blood pressure lowering drugs or if the patient needs antihypertensive treatment to maintain blood pressure under 140/90 mmHg); 2) Blood pressure was higher than 140 (systolic) and/or 90 mmHg (diastolic) in any measurement performed within the first 6 hours after admission. If blood-pressure-lowering agents have been administered before admission and blood pressure measurement is believed to be reliable, a measurement >140/90 mmHg before admission is useful also; 3) The hemorrhage is located deeply (thalamus, putamen, pallidus, caudate, internal capsule, deep white matter, isolated intraventricular hemorrhage, pons, cerebellum); 4) There is no other '3', '2' or '1' etiology. The presence of deep-located microbleeds is considered supportive but not required for the diagnosis.

**Table 1:** Etiologic classification of ICH attributed to Arterial Hypertension.

H score	Prior Arterial Hypertension	Evidence of Arterial Hypertension	Admission Blood Pressure >140/90 mmHg	Deep location	Alternative cause
H_1_	+	+/-	+	+	-
H_1_	-	+	+	+	-
H_2_	+	+/-	+	+	+
H_2_	+	+/-	-	+	-
H_2_	+	+/-	+	-	-
H_2_	-	+	+	+	+
H_2_	-	+	-	+	-
H_2_	-	+	+	-	-
H_3_	+	+/-	+	-	+
H_3_	+	+/-	-	+	+
H_3_	+	+/-	-	-	-
H_3_	-	+	+	-	+
H_3_	-	+	-	+	+
H_3_	-	+	-	-	-
H_3_	-	-	+	+	-
H_3_	-	-	+	+	+

H_2_: Probable hypertensive ICH. The patient had prior arterial hypertension (or it was demonstrated as described above) and has two out of three of the other criteria. H_3_: Possible hypertensive ICH. The patient had prior arterial hypertension (or it was demonstrated as described above) and has one out of three of the other criteria. When there is no information about prior arterial hypertension, but the patient fulfills two or three of the other criteria, the classification is also possible hypertensive ICH.

A (Cerebral amyloid angiopathy, see Table 2). We have adopted the validated Boston criteria for CAA as possible (A_3_), probable (A_2_) and definite (A_1_) CAA [8] with the modification proposed by Linn *et al*. regarding the presence of siderosis [9]. This means that a definite diagnosis (A_1_) can be achieved only with a full postmortem examination. Also, for a patient aged ≥55 with a lobar hemorrhage, at least possible etiology ('A_3_') should be scored (unless “other cause of hemorrhage or superficial siderosis” exists). We require also the absence of any other condition listed in the “Infrequent” category that is clearly related to the current lobar, cortical, or corticosubcortical hemorrhage.

**Table 2:** Classification of ICH attributed to CAA (modified Boston criteria).

CAA score	Postmortem examination^a^	Pathologic tissue (evacuated hematoma or cortical biopsy)	Lobar, cortical, or subcortical hemorrhage	Age ≥ 55y	Other diagnostic lesion (A_1_, A_2a_) Other cause of hemorrhage or siderosis (A_2b_, A_3_)^b^
A_1_	+	+/-	+	+	-
A_2A_ with supporting pathology	-	+	+	+	-
A_2B_	-	-	+^c^ ^or^ ^d^	+	-
A_3_	-	-	+^e^ ^or^ ^f^	+	-

A1: CAA definite

A2A: CAA probable with supporting pathology

A2B: CAA probable

A3: CAA possible

a. Full postmortem examination demonstrating: 1) Lobar, cortical, or corticosubcortical hemorrhage; 2) Severe CAA with vasculopathy; and 3) Absence of other diagnostic lesion

b. Other causes of intracerebral hemorrhage: excessive warfarin (INR>3); antecedent head trauma or ischemic stroke; CNS tumour, vascular malformation, or vasculitis; and blood dyscrasia or coagulopathy. (INR>3 or other nonspecific laboratory abnormalities permitted for diagnosis of possible CAA.) [8]

c. Multiple hemorrhages restricted to lobar, cortical, or corticosubcortical regions (cerebellar hemorrhage allowed)

d. Single lobar, cortical, or corticosubcortical hemorrhage and focal (restricted to 3 or fewer sulci) or disseminated (affecting at least 4 sulci) superficial siderosis

e. Single lobar, cortical, or corticosubcortical hemorrhage

f. Focal (restricted to 3 or fewer sulci) or disseminated (affecting at least 4 sulci) superficial siderosis [9].

T (Tumour, Tables 3 and 4). This etiology is suspected typically by neuroimaging when there is contrast enhancement, when the lesion is heterogeneous and when the amount of edema is higher than expected for the time elapsed from onset to neuroimaging. The presence of metastasis in other organs, the presence of a solid cancer outside the central nervous system and the increase in tumour marker levels should raise clinical suspicion also. The criteria are different for primary tumours or metastatic tumours. The etiology may be delayed in some cases due to the need for a follow-up neuroimaging a few weeks or up to 3 months after onset.

**Table 3:** Classification of ICH attributed to a primary tumour ('T').

Primary tumour score	Suspicious lesion	Positive biopsy	Alternative cause
T_1_	+	+	-
T_2_	+	-	-
T_3_	+	-	+

**Table 4:** Classification of ICH attributed to a metastatic tumour ('T').

Metastatic tumour score	Known primary solid cancer	Number of lesions	Positive biopsy	Alternative cause
T_1_	+	>1	-	-
T_1_	+	1	+	-
T_2_	+	1	-	-
T_2_	+	>1	-	+
T_3_	-	>1	-	-
T_3_	+	1	-	+

Primary tumour: T_1_: A primary tumour as a definite cause of ICH requires: A compatible lesion on neuroimaging; a positive biopsy; absence of an alternative etiology of any degree of certainty. T_2_: A primary tumour as a probable cause of ICH requires: A compatible lesion on neuroimaging; no positive biopsy; the absence of an alternative definite etiology. T_3_: A primary tumour as a possible cause of ICH requires: A compatible lesion on neuroimaging; no positive biopsy; an alternative possible or probable etiology. Metastatic tumour:

Metastatic tumour. T_1_: A metastatic tumour as a definite cause of ICH requires: A primary solid cancer; one lesion with a positive biopsy or more than 1 brain lesion without a positive lesion; absence of an alternative etiology of any degree of certainty. T_2_: A metastatic tumour as a probable cause of ICH requires: A primary solid cancer and 1 brain lesion; no positive biopsy; absence of an alternative definite etiology. T_3_: A metastatic tumour as a possible cause of ICH requires: Either a solid primary cancer with one brain lesion or no primary cancer with more than one brain lesion; no positive biopsy; if there is more than one lesion, no alternative etiology is diagnosed; if there is one lesion together with a known primary cancer, there is an alternative etiology with any degree of certainty.

O (Oral anticoagulants, Tables 5 and 6). The classification distinguishes between ICH caused by antivitamin K (AVK) OA and by new OA (NOAC).

**Table 5:** Classification of ICH attributed to antivitamin K (AVK).oral anticoagulants.

AVK	The patients is receiving AVK	INR	Alternative cause
O_1_	+	≥2	-
O_2_	+	≥2	+
O_2_	+	<2	-
O_3_	+	<2	+

**Table 6:** Classification of ICH attributed to new oral anticoagulants (NOAC).

NOAC	The patients is receiving NOAC	Blood coagulation test altered	Alternative cause
O_1_	+	+	-
O_2_	+	+	+
O_2_	+	-	-
O_3_	+	-	+

For a diagnosis with any degree of certainty of ICH related to AVK a patient must be receiving AVK treatment. The diagnosis is definite (O_1_) when INR is ≥2 and there is no alternative etiology of any degree of certainty. The diagnosis is probable (O_2_) when INR is ≥2 but a possible or probable alternative etiology exists or when INR is <2 and no alternative etiology exists. Finally, the diagnosis is possible (O_3_*)* when INR is <2 and an alternative possible or probable etiology exists.

A patient with a lobar hemorrhage who is receiving AVK has a possible or probable CAA when INR is under 3. Therefore, a patient with a lobar hemorrhage and an INR 2–3 should be classified as A_3_O_2_ (or A_3_O_3_ if INR is <2).

For a diagnosis with any degree of certainty of ICH related to NOAC, a patient must be receiving NOAC treatment. The diagnosis is definite (O_1_) when blood coagulation tests are altered in any way and there is no alternative etiology of any degree of certainty. The diagnosis is probable (O_2_) when the blood coagulation tests are altered in any way and a possible or probable alternative etiology is made, or when coagulation blood tests are normal and there is no alternative etiology. Finally, the diagnosis is possible (O_3_) when blood coagulation tests are normal and an alternative possible or probable etiology exists.

M (arterio-venous Malformation and cavernoma, Table 7). M_1_: The definite diagnosis of vascular malformation requires: The angiographic demonstration of an arterio-venous malformation (with either conventional catheter angiography, CT-angio or MR-angio), or a MRI lesion highly suggestive of a cavernoma (a delayed MRI 1–3 months after ICH may be preferred for a precise diagnosis); absence of an alternative etiology of any degree of certainty.

**Table 7:** Classification of ICH attributed to a vascular malformation (AVM = arterio-venous malformation).

Arteriovenous Malformation score	Angiographic demonstration	Indirect evidence (neuroimaging)	Alternative probable/possible diagnosis
AVM			
M_1_	+	+/-	-
M_2_	+	+/-	+
M_3_	-	+	+/-
Cavernoma	MRI suggestive	MRI non conclusive or not done (but cavernoma is suspected)	Alternative probable/possible diagnosis
M_1_	+	-	-
M_2_	+	-	+
M_3_	-	+	+/-

M_2_: The probable diagnosis of vascular malformation requires: The angiographic demonstration of an arterio-venous malformation (with either conventional catheter angiography, CT-angio or MR-angio), or a MRI lesion highly suggestive of a cavernoma; an alternative possible or probable etiology. M_3_: The possible diagnosis of vascular malformation requires: Indirect neuroimaging data that suggest an arterio-venous malformation, or a MRI lesion that is not conclusive of cavernoma, or MRI is contraindicated although cavernoma is suspected.

I (Infrequent causes of ICH, Table 8). This category includes conditions that may cause an ICH although with a relatively uncommon frequency. The presence of one of these conditions provides a definite diagnosis (I_1_) when there is no alternative etiology. However, the score is probable (I_2_) if an alternative possible etiology exists; and the score is possible (I_3_) when there is an alternative probable etiology. For example a patient with cirrhosis and a platelet count of 10.000 is classified as I_1_ when there is no other etiologic condition, but as M_3_I_2_ if a cavernoma is possible. A patient with a probable arterio-venous malformation that suffers an ICH after cocaine use must be classified also as M_2_I_3_ (but M_1_ in the absence of cocaine use). A patient receiving OAs with a platelet count of 15000 must be classified as O_2_I_3_.

**Table 8:** Classification of ICH attributed to an infrequent disease.

Infrequent cause score	Infrequent cause	Alternative cause
I_1_	+	-
I_2_	+	+ (alternative is possible)
I_3_	+	+ (alternative is probable)

The list of infrequent causes of ICH includes any of the following: Symptomatic intracerebral hemorrhage caused by thrombolytic treatment (intravenous or intra-arterial): a PH1, PH2 or rPH hemorrhage associated with a neurological worsening (increase of at least 4 points on the NIHSS score) within the first 36 hours after treatment; Treatment with heparin. A patient suffers an ICH while receiving intravenous or subcutaneous heparin at full doses and with alteration of blood coagulation tests. For patients receiving low-molecular weight heparin at full anticoagulant dose the etiology is definite when no alternative etiology exists, probable when an alternative possible etiology exists and possible when an alternative probable etiology exists; Treatment with ≥ 2 antiplatelet agents; Intracranial aneurysm (congenital, mycotic, other causes). Requires angiographic demonstration of an aneurysm topographically related to ICH and some amount of associated subarachnoid hemorrhage; Venous angioma and *telangiectasia* (demonstrated by angiography or MRI and topographically related to ICH); dural AV-Fistula (demonstrated by angiography); Cerebral venous thrombosis. Requires angiographic demonstration of thrombosis of a cortical vein or a venous sinus, Cerebral vasculitis (and other intracranial vasculopathies). Requires angiographic demonstration of typical findings or pathological demonstration of an inflammatory vasculopathy or other vasculopathies (vasoconstriction reversible syndrome, dissection, Moya-Moya, Posterior Reversible Encephalopathy Syndrome, Behçet’s disease); Illegal drugs: Requires evidence (often by laboratory tests) of the presence of drugs in blood or urine (ex: cocaine, crack, amphetamine); Conditions associated with hypertensive crisis (tiramine reaction, feocromocitoma, nasal vasoconstrictors, eclampsia, cardiac catheterization, autonomic dysreflexia syndrome in paraplegics, dental extraction…); Hemorrhagic dyathesis. Diagnosis of acquired or congenital diseases that impair coagulation (for example, hemophilia, advanced liver disease with platelet counts under 10.000, platelet diseases with counts under 10.000, afibrinogenemia, disseminated intravascular coagulation…); Alcohol (chronic or acute alcohol abuse >300gr/week in the absence of any other possible, probable or definite cause); Pituitary apoplexy (bleeding into the pituitary gland, usually harboring and adenoma); Hyperperfusion syndrome (after carotid angioplasty, intracranial angioplasty, thromboendarterectomy, heart transplantation, surgery of congenital heart disease, bypass); Miscellaneous (a temporal link is necessary to consider the etiologic role): Migraine, Spät apoplexy (delayed ICH after traumatic brain injury), methanol intoxication, wasp and scorpion bite, trigeminal nerve stimulation, exposure to cold, break-dance, electroshock, and roller coaster).

C (Cryptogenic). A patient with a C-ICH is a patient without a possible, probable or definite etiology of ICH. This category requires that the basic study has been completed. To perform or not an extended diagnostic work-up is at the discretion of the treating physician.

### Application of the H-ATOMIC criteria to a multicenter series of patients with ICH

The local Ethics Committee approved our study in all centers (a waiver was achieved in one of them). A signed consent from each patient or the legal representative was not deemed necessary by the Committee due to the absence of a change in the routine management of the patients and also that the data obtained were anonymous. From July 2013 to December 2014 we prospectively included consecutive patients with ICH who were older than 18 years, and who were admitted to any of the 5 participating tertiary university hospitals during the study period. Patients were usually admitted in any of the following Hospital Departments: Emergency, Intensive Care Unit, Neurology, Neurosurgery, and Internal Medicine. We excluded patients with traumatic ICH. A principal investigator from each center applied the H-ATOMIC criteria to classify the patients.

The following variables were recorded for each patient: 1) Demographic data (age, sex); 2) Vascular risk factors and other risk factors for ICH (blood pressure, diabetes mellitus, hypercholesterolemia, ischemic heart disease, previous ischemic stroke, previous ICH, primary tumour, previous arterio-venous malformation or cavernoma, pre-treatment with antiplatelets and anticoagulants); 3) Prior cognitive impairment; 4) Previous Rankin scale score; 5) Glasgow coma scale score and the National Institute of Health and Stroke (NIHSS) scale score at admission; 6) Neuroimaging variables: hematoma volume at admission (by the ABC/2 method), presence of intraventricular hemorrhage, ICH location, time to baseline CT, SICH score, any angiographic study, MRI; 7) Blood pressure and blood glucose at admission; 8) Surgical evacuation; 9) Rankin scale score at discharge and mortality during hospitalization.

#### Statistical analysis

The results are reported as means and standard deviations for continuous quantitative variables, as median and interquartile range for quantitative ordinal data (NIHSS and GCS scores) and as number and percentages for categorical variables. After receiving training consisting on the application of the H-ATOMIC criteria, a random selection of the discharge reports of 29 patients were reviewed by a group of evaluators consisting of five stroke neurology experts. After revision, consensus was reached for the etiological classification of each patient and the percentage of agreement with the consensus for each evaluator was calculated.

## Results

We studied prospectively 466 consecutive patients. The required basic study was not performed in 27 of them. Our final sample is composed by 439 patients, with a mean age of 70.9 ± 14.6, and 61.3% of them were men. As part of the extended etiologic study an angiographic examination (by either CT-angio, MR-angio or conventional angiography) was performed in 157 (35.7%) patients, MR in 159 (36.2%), delayed neuroimaging in 154 (35%), echocardiography/blood pressure map in 54 (12.3%), drug screen in 23 (5.2%), CSF analysis in 14 (3.1%), biopsy or autopsy examination in 12 (2.7%), cancer screening in 38 (11.6%) and contrast CT, contrast MRI or PET in 21 (4.7%). Table 9 provides the clinical, radiological and prognostic characteristics of our patients.

**Table 9:** Clinical, radiological and prognostic characteristics of patients. Values given in percentages (unless specified).

Variable	
Age (y)	70.8 ± 14.5
Sex (% men)	61.3
Hypertension	75.1
Diabetes Mellitus	21.9
Hyercholesterolemia	37.7
Ischemic heart disease	9.6
Previous ischemic stroke	10.5
Previous ICH	7.3
Known primary tumour	6.6
Known prior arteriovenous malformation or cavernoma	1
Pre-treatment with anticoagulants	17.4
Pre-treatment with any antiplatelet	26.7
Prior cognitive impairment	10.3
Previous Rankin scale 0–2	82.8
Glasgow coma scale at admission, median (percentile 25 and 75), n = 432	15 (12–15)
NIHSS score at admission, median (percentile 25 and 75), n = 378	8 (3–19)
Volume of hematoma at the baseline CT (cc) (n = 343)	34.8 (57.8)
Intraventricular hemorrhage	37.9
Location	
Deep	41.7
Lobar	44.4
Massive	2.5
Cerebellum	5.2
Brainstem	2.7
Multiple	1.1
Primary intraventricular	2.3
Time from onset to baseline CT (min)	510 (1422.7)
SICH score	
0	20.5
1	41.6
2	25.6
3	7.3
4	3.4
5	1.4
6	0.2
Any angiographic study	33.9
MRI	33.7
Systolic blood pressure at admission (mmHg)	169.2 (32.5)
Diastolic blood pressure at admission (mmHg)	89.9 (21.2)
Blood glucose at admission (mg/dl)	138.8 (55.2)
Surgical evacuation	10.2
Rankin at discharge ≤ 2	23
In-hospital mortality	30.4

**Fig 1:**
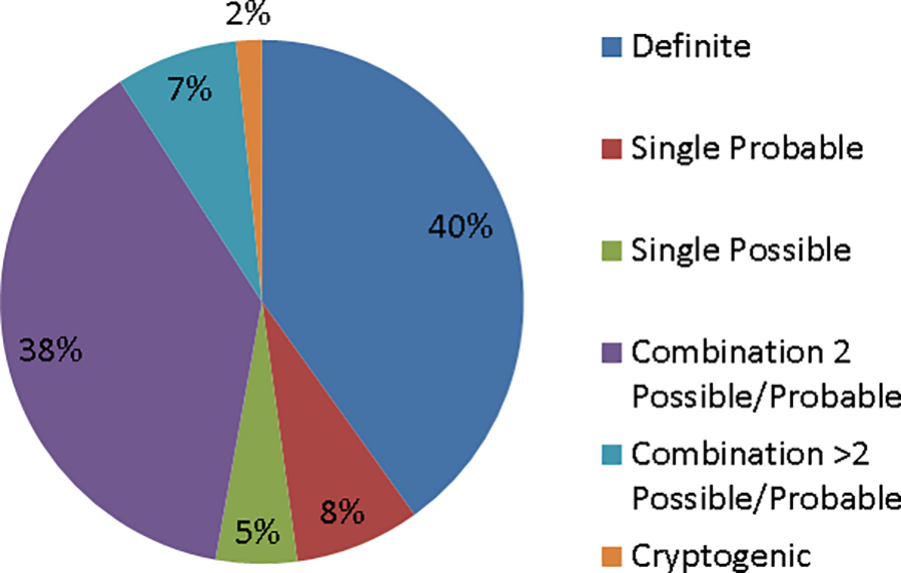
Distribution of etiologic categories in 439 patients (percentages are rounded).

A definite score of etiology was achieved in only 176 (40.1%) patients (Fig 1, S1 Table). A single probable etiology (n = 34, 7.7%) or possible etiology (n = 22, 5%) was scored in 56 (12.7%) patients. An important group (n = 200, 45.5%) had a combination of two (n = 167, 38%) or more (n = 33, 7.5%) etiologies. Finally, 7 patients (1.6%) were classified as cryptogenic.

In patients with a definite etiology, hypertension was the most common etiology (n = 124, 70.5%), followed by vascular malformation (n = 20, 11.4%), infrequent causes (n = 20, 11.4%), OAs (n = 10, 5.7%), while definite CAA (n = 1, 0.5%) and tumour (n = 1 0.5%) were very uncommon. Only 7 patients were classified in the cryptogenic category (1.6%). Infrequent causes consisted of intracranial aneurysms (n = 6), cerebral venous thrombosis (n = 3), intravenous thrombolysis (n = 3), vasculitis (n = 1), posterior reversible encephalopathy syndrome (n = 1), IMAO-related tiramine reaction (n = 1), reversible vasoconstriction syndrome (n = 1), blood dyscrasia (n = 1, von-Willebrand deficiency with factor XI deficiency), ergotic intoxication (n = 1), treatment with antiplatelet agents (n = 1).

The most frequent combinations of etiologies involved possible hypertension with possible CAA (H_3_A_3_, n = 38) or with probable CAA (H_3_A_2_, n = 29), probable hypertension with probable OA (H_2_O_2_, n = 27) and probable hypertension with possible CAA (H_2_A_3_, n = 19). Probable hypertension was a frequent category also (H_2_, n = 25). See S1 Table for details.

**Fig 2:**
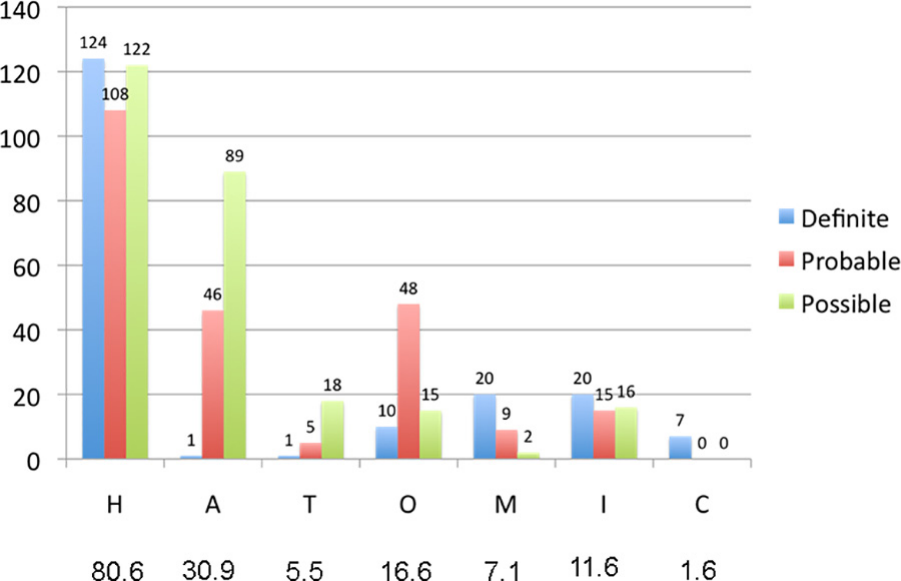
Degree of certainty for each category (n = 439).

Hypertension (Fig 2) was present in all the degrees of certainty (definite, probable or possible) in 354 patients (80.6%); CAA in 136 (30.9%), tumour in 24 (5.4%), OAs in 73 (16.6%), vascular malformation in 31 (7.2%), infrequent causes in 52 (11.8%) and cryptogenic in 7 patients (1.6%).

Percent agreement among stroke neurologists ranged from 72.4 to 96.6%.

## Discussion

H-ATOMIC is the acronym that we propose for a new etiological classification in patients with acute non-traumatic ICH. In a multicentre study we tested this classification system on 439 patients. This classification is based on three assumptions: 1) We propose that the most common etiologies are hypertension (H), CAA (A), tumour (T), OA (O) and vascular malformation (M). There is a long list of infrequent causes (I) that may cause ICH. Also there is the possibility of not finding any etiology (C, cryptogenic ICH). Hence, by necessity, the acronym 'H-ATOMIC'; 2) It is common to find more than one potential etiology for an individual patient. Thus, in addition to patients with a definite etiology, there are patients who have a combination of probable or possible etiologies; 3) The patient with ICH is often unstable during the first hours after onset and, therefore, a basic study designed to find the cause of the ICH must be rapid and easy to perform.

Clinicians use intuitively 4 sources of information to attribute the etiology to a specific disease [2]: Age, location, CT and MRI appearance, and the clinical context (some diseases or risk factors may be typically associated with ICH). However there is no consensus on the diagnostic strategy to be used and on how to combine all this information to reach a final etiologic classification.

Recently, the SMASH-U etiologic criteria [3] were proposed. There are several differences between our proposal and the SMASH-U proposal. In SMASH-U criteria were applied retrospectively to 1013 patients in a single-center; A tumour etiology was not included; More than one etiology was not allowed for a specific patient, and only the most likely cause was considered; No degree of certainty was estimated; No strict definition for each etiology was presented; A list of "other etiologies" was not provided and not defined; Finally, no diagnostic strategy was proposed. A head-to-head comparison between the SMASH-U and H-ATOMIC classification schemes using the same study patients would be needed to allow comparisons of similarities and differences between the two approaches.

According to our H-ATOMIC criteria, a definite etiology was found in only 40.1% of our patients. As expected, arterial hypertension was the most common etiology. It was the cause for 28.2% of the patients from the total sample and of 70.5% of the patients with a definite etiology. Clearly, the importance of hypertension as an etiologic factor is reflected also in its contribution to 80.5% of all patients when including also those patients with possible or probable hypertensive etiology. In the SMASH-U classification[3], 35% of the patients were diagnosed with hypertensive ICH. It is important to emphasize that hypertension is a prevalent condition and when present it does not exclude other etiologies of ICH. For example, hypertension is more frequent in patients with lobar ICH than in the general population [10,11] and is a risk factor also for ICH in patients who are receiving chronic OA [12].

CAA is very difficult to make a definite diagnosis, since it requires pathologic confirmation [8]. As a result, in patients with a lobar ICH over 55 years old and without an alternative explanation, a possible or a probable CAA diagnosis can be made [9, 13]. CAA was related to etiology in 31% of our patients: probable in 20%, possible in 10.5%, and a definite in only 0.5%. This compares with the 20% reported by the SMASH-U study [3]. As a suggestion of relative contribution from different etiologies, it is known that chronic hypertension may facilitate cerebral amyloid deposition [10], and that up to 30% of patients with CAA-related ICH are hypertensives [11, 13, 14, 15]. Amyloid deposition facilitates ICH in patients who receive OA [16].

Either primary or metastatic tumour was uncommon as an etiology of ICH in our study. Tumour etiology was considered with different degrees of certainty in about 5% of our patients.

OA are often a cause of ICH [17, 18]. However, although 73 (17%) of our patients were receiving OA, the cause of hemorrhage was considered definite in only 10 patients (2.3%) and therefore other etiologies must be contributing to the etiology of the remaining 63 patients (in whom OA was a possible or probable etiology). The administration of OA may be complicated by ICH when CAA, hypertension or both coexist in the same patient [12, 16, 19].

Several vascular malformations may cause ICH, but only arterio-venous malformation and cavernoma were considered under our "M" etiology, since other malformations such as aneurysms most frequently present with subarachnoid hemorrhage. The identification of a vascular abnormality is important due to the existence of therapeutic options to prevent recurrence. For this reason, we think it is pertinent to add the SICH score [6] to our classification system. SICH score uses the variables already selected by intuition, as discussed above. Up to 4.5% of our patients had this definite diagnosis, while 2.5% had a probable or possible 'M' diagnosis.

Although it is difficult to separate the relative contribution of an infrequent cause, it was considered to participate in 7% of patients. Finally, only 7 patients (1.6%) were considered truly cryptogenic.

An important finding of our study is that it is difficult to attribute the ICH to a single etiology. It is common (45.5% in our study) to find 2 or more etiologies that may contribute to ICH, with different degrees of certainty for each etiology. Additionally, although in some patients (12.7% in our study) we may identify a single etiology, its degree of certainty is only probable or possible. The H-ATOMIC criteria provide a classification that aims to use all of the available information, by analogy with the A-S-C-O classification of ischemic stroke[20]. For example, in an 80 year-old patient who is hypertensive and is treated with OA and suffers a lobar occipital hemorrhage, it is extremely difficult to know the relative contribution of hypertension, CAA and OA [18]. In the H-ATOMIC classification all the contributing etiologies are considered. For example, 102 patients (23%) in our study had a combination of "H" and "A" etiologies, and sometimes combined with "T", "O" and "I" etiologies, as illustrated in the S1 Table). We believe that selecting a single etiology in these patients loses important information, and neglects the concurrence of more than one etiology of ICH in an individual patient.

ICH is a devastating disease [21]. The patient may develop hemodynamic and cardio-respiratory instability in the early stage and this may interfere with reaching an etiologic diagnosis. So it is prudent to develop a basic diagnostic strategy that is quick and simple. An angiographic analysis, preferably a CT-angiography obtained immediately after the diagnosis, should be made in selected patients in whom the probability of an underlying vascular malformation or venous thrombosis is suspected according to the SICH score [6, 22]. Presently, the resolution of CT-angio is close to conventional angiography and therefore is an excellent screening tool to rule out vascular abnormalities with a sensitivity ≥95% and specificity approaching 100% [23]. Previous studies using conventional catheter angiography yielded few positive diagnoses in patients with previous hypertension and in those with a deep ICH location [2]. An extended diagnostic study may be performed in stable patients hours, days or even weeks after this basic study, depending on the suspicion that arises after a detailed assessment of clinical data and the results of complementary examinations. For this reason, the H-ATOMIC can provide the final classification up to 3 months after the onset of ICH.

Our study has some limitations. This classification system is relatively complex and time-consuming. Also it needs familiarization with the definitions of each etiologic category. Therefore, we are planning to develop an App to be used with the aid of smart phones that would facilitate the etiologic classification process. Finally, we agree that the basic study is too simple and therefore has the risk of loosing important information. Although the study was conducted in tertiary centers, the basic study can also be performed in centers that provide standard stroke care. The recommendation for an extended study for most patients would probably increase the number of patients with a definite score but would also lead to an increase in the number of patients unable to complete the required study. Obviously, a major limitation is the absence of pathologic confirmation for most patients. It is important to obtain histopathological study when patients are operated on or post-mortem. Also, although some ICH topographies may point to an etiology, other locations may be caused by several etiologies.

We strongly believe that there is a need for consensus in classifying the etiology of patients with ICH. This has important implications in our daily care of patients and in designing treatment and research protocols. It is plausible that epidemiology, prognosis, pathogenesis and treatment of sporadic ICH differ among different etiologies. Recent international guidelines [24, 25] do not offer basically any guidance on how to investigate ICH etiology. Hopefully, the prospective application of the H-ATOMIC criteria in a large and different cohort will confirm our results and further refine these criteria.

## Supporting Information

S1 Table List of all etiologic classifications in descending order of frequency. (DOC)
