# Supplementary material for: The H-ATOMIC Criteria for the Etiologic Classification of Patients with Intracerebral Hemorrhage
Source: PLoS One. 2016 Jun 8;11(6):e0156992. doi: 10.1371/journal.pone.0156992 (PMC4898692; doi:10.1371/journal.pone.0156992)
Supplement: S1 Table — (DOC) [file pone.0156992.s001.doc]

Supporting information S1. List of all etiologic classifications in descending order of frequency.

| Etiologic classification | Number of patients | % |
| --- | --- | --- |
| H_1_ | 124 | 28.2 |
| H_3_A_3_ | 38 | 8.7 |
| H_3_A_2_ | 29 | 6.6 |
| H_2_O_2_ | 27 | 6.2 |
| H_2_ | 25 | 5.7 |
| I_1_ | 20 | 4.6 |
| M_1_ | 20 | 4.6 |
| H_2_A_3_ | 19 | 4.3 |
| A_3_ | 11 | 2.5 |
| O_1_ | 10 | 2.3 |
| H_3_ | 9 | 2.1 |
| H_3_I_2_ | 8 | 1.8 |
| C | 7 | 1.6 |
| H_2_O_3_ | 7 | 1.6 |
| H_2_T_3_ | 7 | 1.6 |
| H_3_O_2_ | 7 | 1.6 |
| A_2_ | 6 | 1.4 |
| H_2_A_2_ | 6 | 1.4 |
| H_2_I_3_ | 5 | 1.1 |
| H_3_A_3_T_3_ | 5 | 1.1 |
| H_3_M_2_ | 5 | 1.1 |
| H_3_A_3_I_2_ | 4 | 0.9 |
| H_3_A_3_O_2_ | 4 | 0.9 |
| H_2_A_3_O_2_ | 3 | 0.7 |
| T_2_ | 3 | 0.7 |
| H_3_A_3_O_3_ | 2 | 0.5 |
| H_3_I_3_ | 2 | 0.5 |
| M_3_ | 2 | 0.5 |
| A_1_ | 1 | 0.2 |
| A_3_O_2_I_3_ | 1 | 0.2 |
| H_2_A_2_O_2_ | 1 | 0.2 |
| H_2_A_3_I_3_ | 1 | 0.2 |
| H_2_I_2_ | 1 | 0.2 |
| H_2_O_2_M_2_ | 1 | 0.2 |
| H_2_O_3_I_3_ | 1 | 0.2 |
| H_2_T_2_ | 1 | 0.2 |
| H_2_T_3_I_3_ | 1 | 0.2 |
| H_2_T_3_O_2_ | 1 | 0.2 |
| H_3_A_2_I_3_ | 1 | 0.2 |
| H_3_A_2_O_2_ | 1 | 0.2 |
| H_3_A_2_T_3_O_3_ | 1 | 0.2 |
| H_3_A_3_I_3_ | 1 | 0.2 |
| H_3_O_2_I_2_ | 1 | 0.2 |
| H_3_O_2_M_2_ | 1 | 0.2 |
| H_3_O_3_ | 1 | 0.2 |
| H_3_O_3_I_2_ | 1 | 0.2 |
| H_3_T_3_ | 1 | 0.2 |
| H_3_T_3_M_2_I_3_ | 1 | 0.2 |
| O_3_M_2_ | 1 | 0.2 |
| T_1_ | 1 | 0.2 |
| T_2_O_3_ | 1 | 0.2 |
| T_3_I_2_ | 1 | 0.2 |
| Total | 439 | 100.0 |
